# Evaluation of administrative case definitions for hypertension in Canadian children

**DOI:** 10.1038/s41598-023-33401-x

**Published:** 2023-05-11

**Authors:** Allison Dart, Alexander Singer, Rahul Chanchlani, Thomas Ferguson, Navdeep Tangri, Michael Zappitelli

**Affiliations:** 1 Department of Pediatrics and Child Health, Children’s Hospital Research Institute of Manitoba, Diabetes Research Envisioned and Accomplished in Manitoba, University of Manitoba, FE-009 820 Sherbrook St., Winnipeg, MB R3A1R9 Canada; 2 Division of Family Medicine, University of Manitoba, Winnipeg, Canada; 3 Department of Pediatrics, McMaster University, Hamilton, Canada; 4 Max Rady College of Medicine, Department of Internal Medicine, Chronic Disease Innovation Centre, University of Manitoba, Winnipeg, Canada; 5 Division of Nephrology, Department of Paediatrics, Hospital for Sick Children, University of Toronto, Toronto, Canada

**Keywords:** Nephrology, Paediatrics

## Abstract

Hypertension is increasing in children and warrants disease surveillance. We therefore sought to evaluate the validity of case definitions to identify pediatric hypertension in administrative healthcare data. Cases of hypertension in children 3–18 years of age were identified utilizing blood pressures recorded in the Manitoba Primary Care Research Network (MaPCReN) electronic medical record from 2014 to 2016. Prevalence of hypertension and associated clinical characteristics were determined. We then evaluated the validity of 18 case definitions combining outpatient physician visits (ICD9CM codes), hospital claims (ICD9CM/ICD10 codes) and antihypertensive use within 1–3 years of data housed at the Manitoba Centre for Health Policy. The MaPCReN database identified 241 children with hypertension and 4090 without (prevalence = 5.6%). The sensitivity of algorithms ranged between 0.18 and 0.51 and the specificity between 0.98 and 1.00. Pharmaceutical use increased the sensitivity of algorithms significantly. The algorithms with the highest sensitivity and area under the ROC curve were 1 or more hospitalization OR 1 or more physician claim OR 1 or more pharmaceutical record. Evaluating 2 years of data is recommended. Administrative data alone reflects diagnosis of hypertension with high specificity, but underestimate the true prevalence of this disease. Alternative data sources are therefore required for disease surveillance.

## Introduction

The prevalence of hypertension is estimated to be approximately 3–5%^1–4^ in the general pediatric population in North America and is becoming one of the more common pediatric chronic health conditions. It is increasingly recognized as an important comorbidity of overweight/obesity^5^ and a complication of many chronic health conditions such as chronic kidney disease^6^ and diabetes^7^. Current pediatric studies rely on clinical cohorts^8^, or active surveillance studies^9^ to determine prevalence of pediatric hypertension. These studies are extremely costly and only capture a small proportion of the relevant population.

Administrative health data, which are generated during standard health care delivery have been utilized to evaluate population prevalence of common pediatric health conditions such as diabetes^10^ and inflammatory bowel disease^11^, with support from validation studies that have identified the most reliable case-finding algorithms. Despite these successes, validity studies of case finding for many other health conditions, such as hypertension in children have not been performed^12^. This is likely in large part due to the challenges in obtaining a population-based gold standard comparison group with accurate blood pressures in children.

This study sought to evaluate the validity of administrative data case definitions for hypertension in children utilizing a clinical electronic medical record to identify cohorts with and without hypertension^13^. In addition, we determined the prevalence of hypertension in a population-based sample of children in the Canadian province of Manitoba by age group, the clinical characteristics of children with and without hypertension and their provider type.

## Methods

This is a cross-sectional validation study which utilized the Manitoba Primary Care Research Network (MaPCReN) Electronic Medical Record Databases to develop cohorts of children with and without hypertension utilizing recorded blood pressure standardized to sex and height^13^. The MaPCReN database was then linked to administrative health data housed at the Manitoba Centre for Health Policy to evaluate the validity of 18 case definition algorithms using a combination of outpatient physician, hospital and pharmaceutical data over 1, 2 or 3 years utilizing unique de-identified personal health identification numbers (PHIN)s. Ethics approval was obtained from the Health Research Ethics Board (HREB) at the University of Manitoba (# HS22950 (H2019:249)) and the Health Information Privacy Committee (Project # 2019/20-24). All methods were performed in accordance with the Declaration of Helsinki. Due to de-identified nature of dataset used, need of informed consent was waived by the HREB.

### Data sources

#### Manitoba primary care research network (MaPCReN) electronic medical record databases

This database was used to obtain clinically collected blood pressures for reference standard hypertension diagnosis. It is the Manitoba portion of the Canadian Primary Care Sentinel Surveillance Network (CPCSSN). CPCSSN is a pan-Canadian network that collects national epidemiological data by extracting de-identified patient data from EMR’s of participating primary care providers (family physicians, nurse practitioner and pediatricians). This network has previously been reported to be representative of the general population^14^. Data at the time of the analysis was available for the years 1998–2017.

#### Manitoba health services insurance plan

This database was used to obtain diagnostic codes and pharmaceutical data for the administrative data algorithms. Administrative health data is housed at the Manitoba Centre for Health Policy and contains registration files, physician reimbursement claims (medical services data), hospital discharge abstracts, records of outpatient prescriptions dispensed from the Drug Program information Network (DPIN) as well as laboratory results from Shared Health, Manitoba’s public sector laboratory services provider. Physician reimbursement claims include International Classification of Diseases, 9^th^ Revision, and Clinical Modification (ICD-9CM) diagnostic codes at the 3-digit code level. Hospital abstract data includes ICD-9CM and ICD-10 Canadian version (ICD-10CA) codes depending on the year. The data is stored in de-identified form for research purposes in the form of a Population Health Research Data Repository and linked between databases utilizing a scrambled personal health identification number (PHIN) at the person level by cross-walk file. In order to preserve patient anonymity small cell sizes < n = 6 are required to be suppressed in publications to align with local privacy legislation.

In Canada, there is universal coverage to all Canadian citizens. Public funding is administered on a provincial basis, within the guidelines set by the Canadian Government. In Manitoba, pharmaceutics are covered by a program called Pharmacare after an income-based deductible is met. Private insurance plans are also available.

### Definitions

The MaPCReN database was utilized to develop cohorts of children with and without hypertension between Jan 1, 2014 and Dec 31, 2016. Children with at least 2 blood pressures available for analysis during the period of study (1, 2 or 3 years), were stratified by their hypertension status into 2 cohorts. While the current AAP guidelines suggest an average of 2 blood pressures on a given day be utilized the classify blood pressure^15^, it was rare that 2 readings were available on a given day.

### MaPCReN cohorts (gold standard)

#### Hypertension cohort

##### Inclusion criteria

Children 3 to < 13 years of age with ≥ 2 abnormal blood pressures (> 95th%ile for age, sex and height) based on the clinical standard at that time period (i.e. 4th Report criteria)^13^ and children ≥ 13 years with ≥ 2 blood pressures > 130 systolic OR > 80 diastolic were considered hypertensive. In addition, children 3–18 years who were prescribed treatment with an anti-hypertensive medication were also classified for hypertension if they did not have the following diagnoses in their EMR Problem List: Migraine, Congestive Heart Failure, Myocardial Infarction, Cardiac Arrhythmia, Tremor, Esophageal Varices, Angina, Kidney Stones or Portal Hypertension. These conditions were selected as several common anti-hypertensive medications used in children are known to be used for the treatment of these conditions. Hypertension status was determined over the relevant time period of study (i.e. 1, 2 or 3 years).

##### Exclusion criteria

 Children < 13 years without an available height or sex were excluded as their blood pressure status could not be determined. As 3 years of data were required, we excluded children < 3 years of age during the study period. Children without an available scrambled PHIN were also excluded as they could not be linked with the administrative databases.

#### Normotensive cohort

Children 3–18 years of age with 2 blood pressures available were classified as normotensive if they did not meet criteria for hypertension as described above based on the relevant period of study.

### Clinical characteristics and comorbidities

Age, sex, socioeconomic status by area level income quintile^16^ and BMI-z score were determined. Overweight was defined as BMI z-core > 1 + SD above the mean and obesity > 2 + SD above the mean^17^. Diabetes was defined with the CPCSSN definition (2 ICD codes for diabetes within 2 years OR diabetes in Problem List or 1 prescription for diabetes medication (ATC code A10) OR 2 A1c’s > 6.5% within 1 year^18^. Chronic Kidney Disease was defined as a CKID Schwartz^19^ eGFR < 75 ml/min/1.73 m^2^ and/or urine albumin:creatinine ratio ≥ 3 mg/mmol on 2 occasions at least 90 days apart (as determined from the Shared Health Laboratory database. We also evaluated what proportion of each group was seen by a family physician, pediatrician or nephrologist during the study period.

### Administrative data definitions

The following ICD codes were utilized to identify children with hypertension.

- Physician Claims: (ICD 9CM): 401 (Essential hypertension), 402 (Hypertensive Heart Disease), 403 (Hypertensive Chronic Kidney Disease), 404 (Hypertensive Health and Chronic Kidney disease), 405 (Secondary Hypertension).
- Hospital Claims: (ICD10CA): I10 (Essential hypertension), I11 (Hypertensive heart disease), I12 (Hypertensive renal disease), 13 (Hypertensive heart and renal disease), I15 (Secondary hypertension).
- Pharmaceuticals: Antihypertensive drugs, diuretics, beta blocking agents, calcium channel blocking agents, agents acting on the renin-angiotensin system, or terazosin (Supplemental Table I for full list). Clonidine was excluded due to the common indication for hyperkinetic disorders of childhood.

### Validation methods and analysis

Combinations of healthcare data were used to create 18 different case definitions for evaluation. The case definitions developed included data captured over 1, 2, or 3 years from a combination of 1 or more hospital discharge diagnoses, 1 or 2 or more physician reimbursement claims (medical services data), and/or 1 or 2 or more records of outpatient prescriptions dispensed from DPIN (Table 3). This type of validation analysis has been performed in other validation studies^20^. We also performed a sensitivity analysis excluding the hospitalization data.

## Results

### Cohort characteristics and prevalence of hypertension in the population studied

**Figure 1:**
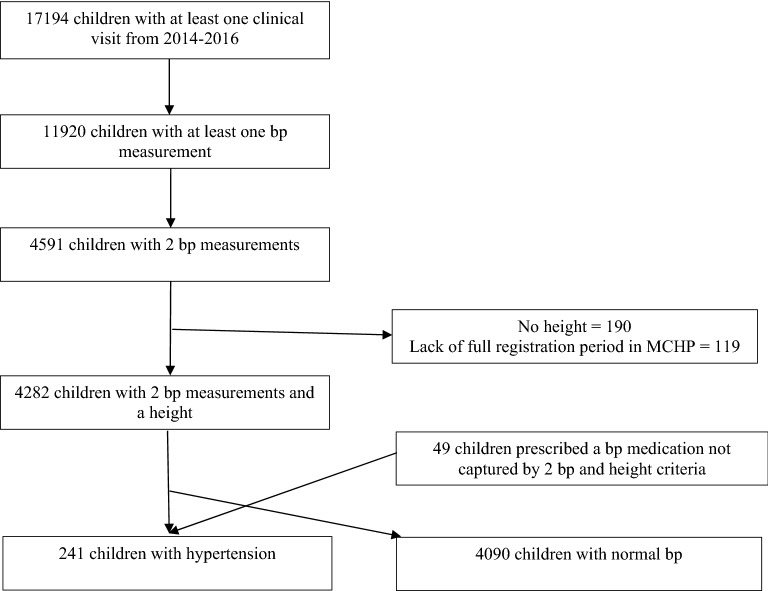
Flow diagram for creation of cohorts of children with and without hypertension.

A total of 192 children were identified with prevalent hypertension based on recorded blood pressures and an additional 49 met criteria based on antihypertensive prescription. An additional 4090 children had normal blood pressure. A total of 17,194 children had at least 1 clinical visit during the study period, however only 4591 had 2 blood pressure measurements recorded (26.7%). There were 190 children < 13 years without an available height and 119 lacked complete registration data in the Manitoba Centre for Health Policy database that were excluded (Fig. 1).

The characteristics of the 2 cohorts are presented in Table 1. Children with hypertension were older (13.7 vs. 10.7 years; p ≤ 0.01) and were more likely to be overweight or obese, have diabetes and have a lower income quintile. We had to suppress the CKD variable as the sample size was < 6/group (to protect anonymity). There was not a difference in prevalence of hypertension according to biologic sex in this population. A total of 8.7% of the hypertension cohort had visited a nephrologist in comparison with 0.8% of the no hypertension cohort.

**Table 1:** Clinical Characteristics of Cohorts of Children with and without Hypertension identified in the Manitoba Primary Care Research Network (MaPCReN).

	Hypertension	No hypertension	p-value
	n = 241	n = 4090	
Age, mean (SD)	13.7 ± 3.9	10.7 ± 4.2	< 0.01
Female (%)	140 (58.1%)	2131 (52.1%)	0.07
BMI (kg/m^2^), mean (SD)	26.2 ± 8.3	19.3 ± 5.0	< 0.01
BMI-Z, median (IQR)	1.44 (0.29–2.21)	0.35 (-0.39–1.13)	< 0.01
Weight category			< 0.01
Normal/thin	138 (57.3%)	2963 (72.4%)	
Overweight	45 (18.7%)	808 (19.8%)	
Obese	58 (24.1%)	319 (7.8%)	
Income quintile			< 0.01
Q1	45 (19.2%)	440 (11.0%)	
Q2	46 (19.7%)	619 (15.4%)	
Q3	40 (17.1%)	693 (17.3%)	
Q4	40 (17.1%)	808 (20.1%)	
Q5	63 (26.9%)	1451 (36.2%)	
Diabetes	9 (3.7%)	36 (0.9%)	< 0.01
Visited family physician	231 (95.9%)	3377 (82.6%)	< 0.01
Visited pediatrician	87 (36.1%)	2862 (70.0%)	< 0.01
Visited nephrologist	21 (8.7%)	33 (0.8%)	< 0.01

Table 2 shows the prevalence of hypertension based on 1 year, 2 years or 3 years of data by age group. The overall prevalence ranged from 5.6 to 5.8% and increased from < 3% in 3–5 year age group, 2.5–4.8% in 6–12 year and 10.1–17.0% in 13–18 year age group.

**Table 2:** Prevalence of pediatric hypertension based on years of data evaluated and age group.

1 year (2016)	Children with hypertension (n (%))	Total sample available (n = 888)
Age 1–5 years	< 5	62
Age 6–12 years	20 (4.8%)	415
Age > 12 years	70 (17.0%)	411

2 years (2015–2016)		n = 2821
Age 1–5 years	9 (2.4%)	378
Age 6–12 years	34 (2.5%)	1380
Age > 12 years	120 (11.3%)	1063

3 years (2014–2016)		n = 4331
Age 1–5 years	15 (2.9%)	515
Age 6–12 yeard	55 (2.6%)	2130
Age > 12 years	171 (10.1%)	1686

### Case identification by administrative data

Table 3 includes the number of children identified with each case definition algorithm as well as those identified in the electronic medical record and both data sources. In general, more children were identified with increasing years of data available. The total number of children that met criteria for hypertension within 1 year of data were relatively small (total n ranging between 94 and 111 depending on the algorithm and data source), which increased to 175–194 for 2 years of data, and 261–289 for 3 years data. If the EMR data was not available, then the number of children identified with admin data would have been ≤ 65 during a 1-year period, 43–100 during a 2-year period and 66–142 over 3 years. The inclusion of pharmaceutical data increased number of children identified by administrative data by 2–3-fold. A minority of cases were identified in the administrative data only, without being identified as hypertensive in the EMR reference standard (< 5.3–16.6%).

**Table 3:** Case definitions for hypertension in children 3–18 years of age and number of children identified by administrative data, MaPCReN electronic medical record (EMR) data or both data sources.

Years of data collection	Algorithm	Hospital separations	Physician claims	Pharmaceutical records	Administrative data base only	EMR only	Administrative data and EMR
1 2016	1	1 or more	1 or more		8	71	23
	2	1 or more	2 or more		< 5	77	17
	3	1 or more	1 or more	1 or more	17	46	48
	4	1 or more	2 or more	1 or more	15	48	46
	5	1 or more	1 or more	2 or more	15	53	41
	6	1 or more	2 or more	2 or more	13	58	36
2 2015/2016	7	1 or more	1 or more		17	124	39
	8	1 or more	2 or more		12	132	31
	9	1 or more	1 or more	1 or more	31	94	69
	10	1 or more	2 or more	1 or more	27	99	64
	11	1 or more	1 or more	2 or more	26	103	60
	12	1 or more	2 or more	2 or more	22	108	55
3 2014/2015/2016	13	1 or more	1 or more		29	187	54
	14	1 or more	2 or more		20	195	46
	15	1 or more	1 or more	1 or more	48	147	94
	16	1 or more	2 or more	1 or more	39	153	88
	17	1 or more	1 or more	2 or more	44	161	80
	18	1 or more	2 or more	2 or more	35	167	74

Administrative data = medical claims (physician visits), discharge abstracts (hospitalizations), pharmaceutical records; MaPCReN = Electronic Medical Record data (reference standard).

### Validation results for administrative data algorithms

Table 4 includes the validation results for all 18 case definition algorithms. The sensitivity ranged between 0.18 and 0.51 (low-modest) but the specificity was very high, between 0.98 and 1.00. The positive predictive values were modest 0.65–0.77 and negative predictive values were excellent between 0.91 and 0.97. The area under the receiver operating curves were 0.59–0.74 and kappa coefficients were 0.26–0.57, demonstrating a low-moderate level of agreement.

**Table 4:** Validation results for case definitions for hypertension in children 3–18 years of age.

Years of data	Algorithm	Hospital separations	Physician claims	Drug records	Sensitivity	Specificity	Positive predictive value	Negative predictive value	Kappa	Area under Receiver operating curve
1 (2016) N = 888	1	1 or more	1 or more		0.24 (0.16–0.33)	0.99 (0.98–1)	0.74 (0.59–0.9)	0.92 (0.9–0.94)	0.33 (0.23–0.44)	0.62
	2	1 or more	2 or more		0.18 (0.1–0.26)	0.99 (0.99–1)	0.77 (0.6–0.95)	0.91 (0.89–0.93)	0.26 (0.16–0.37)	0.59
	3	1 or more	1 or more	1 or more	0.51 (0.41–0.61)	0.98 (0.97–0.99)	0.74 (0.63–0.85)	0.94 (0.93–0.96)	0.57 (0.47–0.66)	0.74
	4	1 or more	2 or more	1 or more	0.49 (0.39–0.59)	0.98 (0.97–0.99)	0.75 (0.65––0.86)	0.94 (0.93–0.96)	0.56 (0.46–0.65)	0.74
	5	1 or more	1 or more	2 or more	0.44 (0.34–0.54)	0.98 (0.97–0.99)	0.73 (0.62–0.85)	0.94 (0.92–0.95)	0.51 (0.41–0.61)	0.71
	6	1 or more	2 or more	2 or more	0.38 (0.28–0.48)	0.98 (0.97–0.99)	0.73 (0.61–0.86)	0.93 (0.91–0.95)	0.46 (0.36–0.57)	0.68
2 2015/16 N = 2821	7	1 or more	1 or more		0.24 (0.17–0.3)	0.99 (0.99–1)	0.7 (0.58–0.82)	0.96 (0.95–0.96)	0.34 (0.26–0.42)	0.62
	8	1 or more	2 or more		0.19 (0.13–0.25)	1 (0.99–1)	0.72 (0.59–0.85)	0.95 (0.94–0.96)	0.28 (0.2–0.36)	0.59
	9	1 or more	1 or more	1 or more	0.42 (0.35–0.5)	0.99 (0.98–0.99)	0.69 (0.6–0.78)	0.97 (0.96–0.97)	0.5 (0.43–0.58)	0.71
	10	1 or more	2 or more	1 or more	0.39 (0.32–0.47)	0.99 (0.99–0.99)	0.7 (0.61–0.8)	0.96 (0.96–0.97)	0.48 (0.41–0.56)	0.69
	11	1 or more	1 or more	2 or more	0.37 (0.29–0.44)	0.99 (0.99–0.99)	0.7 (0.6–0.79)	0.96 (0.96–0.97)	0.46 (0.38–0.54)	0.68
	12	1 or more	2 or more	2 or more	0.34 (0.26–0.41)	0.99 (0.99–1)	0.71 (0.61–0.82)	0.96 (0.95–0.97)	0.44 (0.36–0.52)	0.66
3 14/15/16 N = 4331	13	1 or more	1 or more		0.22 (0.17–0.28)	0.99 (0.99–1)	0.65 (0.55–0.75)	0.96 (0.95––0.96)	0.31 (0.25–0.38)	0.61
	14	1 or more	2 or more		0.19 (0.14–0.24)	1 (0.99–1)	0.7 (0.59–0.81)	0.95 (0.95–0.96)	0.28 (0.22–0.35)	0.59
	15	1 or more	1 or more	1 or more	0.39 (0.33–0.45)	0.99 (0.98–0.99)	0.66 (0.58–0.74)	0.96 (0.96–0.97)	0.47 (0.41–0.53)	0.69
	16	1 or more	2 or more	1 or more	0.37 (0.3–0.43)	0.99 (0.99–0.99)	0.69 (0.61–0.77)	0.96 (0.96–0.97)	0.46 (0.39–0.52)	0.68
	17	1 or more	1 or more	2 or more	0.33 (0.27–0.39)	0.99 (0.99–0.99)	0.65 (0.56–0.73)	0.96 (0.96–0.97)	0.42 (0.35–0.48)	0.66

The algorithm with the highest sensitivity (0.51; 95% CI 0.41–0.61) and area under the ROC (0.74) was #3 which included 1 or more hospitalization OR 1 or more outpatient physician claim OR 1 or more anti-hypertensive. The sensitivity analysis which removed the option for 1 or more hospitalization over 1 year revealed a similar sensitivity of 0.51 (95% CI 0.42–0.61). The algorithm # 9 which included 2 years of data had a sensitivity of 0.42 (95% CI 0.35–0.5) and an area under ROC of 0.71. The sample available for the validation analysis increased from 888 in 2016 to 2821 in 2015–2016.

## Discussion

In this population-based cohort study, we identified the requirement of 1 or more hospitalization OR 1 or more outpatient visit OR 1 or more prescriptions for an anti-hypertensive medication as the case definition algorithm with the highest sensitivity and specificity for the diagnosis of pediatric hypertension utilizing administrative healthcare data. In general, algorithms had relatively modest sensitivity, improved positive predictive value and excellent specificity and negative predictive value for pediatric hypertension. To our knowledge, this is the first study to evaluate administrative healthcare data algorithms for children, and therefore addresses an important knowledge gap in this evolving area of population health research.

Previous studies in adults support the use of administrative health data for disease surveillance. Administrative data is collected in real time, captures the majority of individuals receiving medical care and therefore reflects near-population prevalence of disease, with few limitations. In universal health care systems, like in Canada or several European countries, administrative data can accurately capture trends in incidence and prevalence of chronic conditions and outcomes over time. The national Canadian Chronic Disease Surveillance System (CCDSS) has been developed utilizing administrative health data to evaluate trends for over 20 chronic health conditions including hypertension^21^. Until now, children have been excluded, likely due to a lack of validation studies.

A recent systematic review has been published summarizing validation studies for hypertension in adults in 5 Canadian provinces^22,23^. The sensitivity of the standard definition which includes 2 outpatient physician claims within a 2- year period or 1 hospitalization is 71.2% (95% CI 68.3–73.7) and the specificity is 94.5% (95% CI 93.2–95.6). Gold standard cohorts for the included studies included self-reported data from the Canadian Community Health Survey^24^ and chart reviews^25^. There was substantial agreement between reference standards in all studies. In these studies, a decrease in the time frame to 1 year decreased sensitivity, while increasing the time frame increased sensitivity slightly, but decreased specificity. The removal of hospitalization data resulted in a slightly lower sensitivity. They did not evaluate the utilization of drug data in these studies.

In contrast to adult hypertension, which has been shown to be decreasing in disease surveillance studies^21^, pediatric hypertension is increasing in children and now occurs in up to 5% of the general pediatric population^4^, in keeping with our findings. It has been shown to track into adulthood^26^ and is associated with target-organ damage including left ventricular hypertrophy^27^ and early evidence of atherosclerotic disease. Children with overweight/obesity, and other chronic health conditions such as diabetes are at particularly high risk^28^. As the rates of these health conditions increase^29^, so too will rates of hypertension. Developing chronic disease surveillance strategies that include children should be the standard moving forward.

This study highlights that hypertension remains underdiagnosed in primary care settings. Only 26.7% of the population captured had a blood pressure available for assessment. In addition, only 8.7% of children with hypertension had a nephrology visit in a 3-year time period. The lack of blood pressure screening has been previously identified as an important issue and continues to be exacerbated by conflicting guideline recommendations^30^. Pediatric expert consensus guidelines clearly recommend yearly screening and treatment of all children 3 years of age and up^28^, whereas the US Preventive Services Task Force states there is insufficient evidence to recommend it^31^. On a positive note, recent American^28^ and Canadian guidelines^32^ have sought to decrease the complexity of diagnosis and treatment thresholds, and efforts are underway to translate knowledge to primary care practitioners. This study should be repeated to evaluate the validity of administrative data once screening and management guidelines have been more broadly implemented.

Our study has several strengths and some limitations. First, the Manitoba Centre for Health Policy database has allowed evaluation of hospital, physician and drug data which has identified the importance of pharmaceutical data to identify children with hypertension with administrative data. We identified a population-based sample of children from the primary care setting with and without hypertension to serve as the reference standard utilizing real-world blood pressures stratified by standards at the time. As this cohort reflects a real-world clinical population, we must acknowledge there is not an optimal number of blood pressure readings available, reflecting clinical practice^33^. However, due to the challenges obtaining a population-based sample for this type of study, the requirement of at least 2 abnormal blood pressures is a pragmatic sample for a pediatric hypertension cohort. As there were over 4000 children captured with normal blood pressures, and 2 abnormal readings were required to classify hypertension, the likelihood of false negatives and positives is low. A population-based sample with 24-h ambulatory blood pressures available is not practical. Another issue is that the sample available for 1 year of data is likely inadequate to reliably evaluate validity characteristics. For this reason, the authors suggest utilizing 2 years of data to evaluate children with hypertension despite the slightly higher sensitivity of 1 year of data collection. As with all administrative data studies there are additional limitations including the ability of physicians to only record 1 disease per healthcare encounter, thereby potentially limiting the capture of hypertension as a comorbidity in some cases.

In conclusion, this study has evaluated the validity of administrative data to identify children with hypertension based on pediatric standards. It has identified a modest sensitivity, and excellent specificity for diagnosis of hypertension in children. There is a clear need to repeat this study in the future to re-evaluate case finding with simplified blood pressure standards for children. The concomitant use of electronic medical records may be required to adequately perform disease surveillance in the current landscape.

## Supplementary Information

Supplementary Information.

## Data Availability

Data used in this article was derived from administrative health and social data as a secondary use. The data was provided under specific data sharing agreements only for approved use at Manitoba Centre for Health Policy (MCHP). The original source data is not owned by the researchers or MCHP and as such cannot be provided to a public repository. The original data source and approval for use has been noted in the acknowledgments of the article. Where necessary, source data specific to this article or project may be reviewed at MCHP with the consent of the original data providers, along with the required privacy and ethical review bodies. For more information please contact: mchp_access@cpe.umanitoba.ca.
