# Supplementary material for: Evaluation of administrative case definitions for hypertension in Canadian children
Source: Sci Rep. 2023 May 11;13:7654. doi: 10.1038/s41598-023-33401-x (PMC10175274; doi:10.1038/s41598-023-33401-x)
Supplement: Supplementary file 1 — Supplementary Information. [file 41598_2023_33401_MOESM1_ESM.docx]

Supplemental Table I List of ICD codes and pharmaceuticals evaluated. Bolded drugs were identified in pharmaceutical database.

| ICD 9CM Codes | ICD 10 Codes | Pharmaceuticals |
| --- | --- | --- |
| 401 – Essential hypertension  402 – Hypertensive heart disease  403 – Hypertensive nephropathy  404 –Hypertensive cardionephropathy  (437 –Hypertensive encephalopathy)  405 – Secondary hypertension | I10-Essential Hypertension  I11 Hypertensive Heart Disease  I12 Hypertensive CKD  I13 Hypertensive heart and CKD  I15 Secondary Hypertension  (I14 does not seem to exist) | Antihypertensives:  **C02AB01/02 – methyldopa**  C02CA04 – doxazosin  C02CA05 – terazosin  **C02DB02 – hydralazine**  C02DC01 – minoxidil  C02LA01 – reserpine + diuretics  C02LB01 – methyldopa and diuretics  Diuretics:  **C03AA03 – hydrochlorothiazide**  C03BA04 – chlorthalidone  C03BA11 - indapamide  C03CA01 – furosemide  C03CA02 – bumetanide  C03CC01 – ethacrynic acid  C03DA01- spironolactone  C03DB01 - amiloride  C03DB02 – triamterene  C03EA01 – hctz + K sparing diuretic  Beta Blocking Agents:  C07AA02 – oxprenolol  C07AA03 – pindolol  C07AA05 – propranolol  C07AA06 - timolol  C07AA12 – nadolol  **C07AB02 – metoprolol**  **C07AB03 – atenolol**  C07AB04- acebutolol  **C07AB07-bisoprolol**  **C07AG01 - labetolol**  C07BA05 – propranolol and thiazides  C07BA06 – timolol and thiazides  C07CA03- pindolol + other diuretics  C07CB03 – atenolol + diuretic  Calcium Channel Blockers:  **C08CA01 – amlodipine**  C08CA02 – eposartan  C08CA04- Irbesartan  **C08CA05 – nifedipine**  C08CA06- nimodipine  C08DA01 – losartan and diuretics  C08DB01 – diltiazem  Agents Acting on the RAS:  C09AA01- captopril  **C09AA02- enalapril**  **C09AA03 – lisinopril**  **C09AA04 – perindopril**  C09AA05 – ramipril  C09AA06 – quinapril  C09AA07-benazepril  C09AA08- cilazapril  **C09AA09 – fosinopril**  C09AA10-trandolapril  **C09BA02 -enalapril + diuretics**  **C09BA03 – lisinopril + diuretics**  **C09BA04 – perindopril + diuretics**  C09BA06 – quirapril + diuretics  C09BA08 – cilazapril + diuretics  C09BA10 – trandolapril + diuretics  **C09CA01- losartan**  C09CA02 - eprosartan  C09CA03-valsartan  **C09CA04- irbesartan**  **C09CA06 - candesartan**  C09CA07- telmisartan  C09CA08-olmesartan  C09DA01-losartan + diuretics  C09DA02- eprosartan + diuretics  C09DA03- valsartan + diuretics  C09DA04- ibesartan + diuretics  C09DA06 – candesartan + diuretics  C09DA07 – telmisartan + diuretics  C09DA08 – Olmesartan + diuretics  C09DB02 – Olmesartan + medoxomil + amlodipine  C09XA02 - aliskiren  C09XA52 – aliskiren + diuretics  Other:  C10BX03 – atorvastatin + amlodipine  G04CA03 – terazosin |
